# Match Performance Analysis of Women’s Épée in the 2017–2019 World Fencing Championships

**DOI:** 10.5114/jhk/203422

**Published:** 2025-09-23

**Authors:** Jo-Ting Hsu, Yin-Hua Chen

**Affiliations:** 1 Graduate Institute of Athletics and Coaching Science, National Taiwan Sport University, Taoyuan, Taiwan.

**Keywords:** attack, counterattack, notational analysis, piste, scores

## Abstract

Épée preserves the original dueling nature of fencing and is the only discipline that allows double hits. However, there is still limited understanding of how specific techniques are used in different contexts, such as bout rounds, piste areas, and score statuses (leading, tied, or trailing). To address this gap, this study analyzed the performance of elite female épée fencers during the top 16 to final bouts of the 2017–2019 world championships using notational analysis of videos from the International Fencing Federation’s official YouTube channel. This dataset included 1,840 scoring events across 45 matches, involving 38 fencers. Three- or two-way mixed-design ANOVAs, as well as two-way repeated measures ANOVAs were conducted to investigate the effects of these factors and their interactions for both winners and losers. Results highlighted the second round as a crucial scoring phase. Winners strategically focused on achieving more single hits (than double hits), attacking in the first two rounds and counterattacking in the third. When leading, winners often lured their opponent toward their side of the piste to maintain control, while becoming more aggressive—advancing into the central or even the middle area of the opponent’s side to score with attacks—when temporarily tied or trailing. In contrast, losers frequently scored in the central and middle areas of both sides. When temporarily leading, they displayed no clear preference for specific techniques or areas of the piste. These findings provide valuable insights for coaches to design more effective training sessions and offer targeted feedback during competitions to enhance fencers’ performance.

## Introduction

Fencing is an open-skilled combat sport. In modern competitive fencing, the sport is divided into three disciplines: épée, foil, and saber, with both male and female competitions. Each discipline is defined by specific rules governing techniques and target areas (for a review, see Roi and Bianchedi, 2008; the latest updates please refer to FIE, 2023). The use of a foil and a saber is regulated by established rules, including priority rules, to determine which fencer’s hit takes precedence when simultaneous hits occur. In contrast, épée is the only one that preserves the original dueling nature and permits double hits (Roi and Bianchedi, 2008). According to material rules of FIE (2023), a minimum pressure of 750 g (with a tolerance of ± 3 g) fully applied to the tip of the sward for 2–10 ms can be registered as a hit on the scoring machine. Double hits are allowed when the two hits occur within 40 ms of each other.

The field of play for fencing competition is called a piste. According to technical rules of FIE (FIE, 2023), it is 1.5-m to 2-m wide and 14-m long. It features two on-guard lines, positioned 2 m from a center line, where fencers begin their bout. Moreover, there are two warning lines, located 2 m from either end (marked as rear limit lines), which help fencers keep track of their position on the piste. In an individual match, fencers can win by being the first to score 5 points in preliminary bouts or 15 points in direct elimination bouts against their opponent. Alternatively, they can secure victory by having a higher score than their opponent when the duration of the bout expires, which is 3 min for preliminary bouts and 9 min (organized into three 3-min periods with 1-min break in between) for direct elimination bouts. Note that duration of the bout is held to mean “effective duration”, that is the total of the time intervals between the orders “Play!” and “Halt!” controlled by a referee with a timekeeper. If no winner emerges following this process, an additional 1-min sudden death overtime is granted, and the fencers fence for a deciding hit. Before the fencing recommences the referee draws lots to decide who will be the winner if scores are still equal at the end of the extra minute (FIE, 2023).

The application of sports performance analysis, encompassing both match analysis and time-motion analysis of videotaped matches, is employed to scrutinize the actions performed during a match (Hughes and Franks, 2007; O'Donoghue, 2014). Match analysis focused on evaluating the effectiveness of techniques, tactics, and decision-making, while time-motion analysis provides insights into the physical demands whether for an individual player as well as the entire team (Hughes and Franks, 2007; O'Donoghue, 2014; for a review in fencing, see Turner et al., 2014). For example, Roi and Pittaluga (1997) conducted a time-motion analysis involving 21 bouts at an unspecified competition level from 42 women épée fencers of varying skill levels. The results revealed that the average time for each action was 16.5 ± 4.2 s, while interruption averaged 7.9 ± 2.7 s, leading to a work-to-recovery (action-to-interruption) ratio of 2:1. Furthermore, fencers with higher technical proficiency displayed more frequent changes in movement direction (forward-backward and vice versa) than their less-skilled counterparts (133 ± 62 vs. 85 ± 25), confirming that a higher number of directional changes is an indicator of enhanced performance (Roi and Pittaluga, 1997). On the other hand, Wylde and colleagues (2013) conducted a time-motion analysis on elite woman foil fencers based on 100 international fencing bouts. They reported a work-to-recovery ratio of 1:1.4 for 15-touch bouts and 1:1 for 5-touch and team bouts. Consequently, they proposed that identical training plans could be employed to physically prepare fencers for 15-touch, 5-touch, and team bouts (Wylde et al., 2013).

There was also one study in saber in which Aquili and colleagues (2013) analyzed 35 men’s and 25 women’s saber bouts during 2009–2010 world cup competitions (FIE GP and A). Their results revealed that the work-to-recovery ratio was 1:6.5 for men and 1:5.1 for women. The majority of actions in those boults were offensive (55% for men and 49% for women), with the central 4 m of the piste being predominantly utilized (72% for men and 67% for women). Moreover, the differences between male and female saber fencers were significant. Compared to previous studies involving épée and foil fencers (Roi and Pittaluga, 1997; Wylde et al., 2013), saber fencers exhibited shorter action duration, a higher frequency of offensive actions, and a greater number of actions in the central 4 m of the piste, requiring rapid acceleration and deceleration actions. The authors thus concluded that saber was a fencing discipline characterized by speed and instinct, distinguishing it from épée and foil (Aquili et al., 2013).

More recently, Tarrago et al. (2023) conducted a time-motion analysis during 2014 fencing world championships that involved 96 elite fencers for the three weapons participating in 83 bouts. The work-to-rest ratios were 1:0.9, 1:2.6, and 1:9.2 for épée, foil and saber, respectively, with significant differences among the three disciplines, but no gender differences within the same discipline. The incongruence with previous studies (Aquilli et al., 2013; Roi and Pittaluga, 1997; Wylde et al., 2013) was mainly attributed to variations in the rules. For example, in the study of Roi and Pittaluga (1997), bouts were limited to 10 hits and had duration of 10 min in épée. Moreover, there were differences in the required time for a double hit in saber, that was 400 ms in the 2019–2010 season (Aquilli et al., 2013), but 130 ms in the 2014 season (Tarrago´ et al., 2023). Tarrago´ et al. (2023) concluded that there was a greater reliance on the alactic energy system in saber compared to épée and foil even though previous studies often suggested that all three disciplines rely on the alactic energy system to provide explosive movements like a lunge (Bottoms et al., 2011; Oates et al., 2019; Turner et al., 2014). The findings from studies using time-motion analysis can be used for improving or designing specific training sessions (Hughes and Franks, 2007; O'Donoghue, 2014; Turner et al., 2014).

Although time-motion studies have updated the physical demands for the three fencing disciplines in recent decades, there remains a gap in understanding how specific techniques are used in varying contexts. According to the Newell’s constraints model (1986), movement coordination is shaped by the individual, task, and environmental constraints. Contextual factors such as bout rounds, piste areas, and score statuses (leading, tied, or trailing), can act as task constraints, influencing how fencers apply their techniques. The most related study we found was conducted by Zadorozhna and colleagues (2018) who investigated the individual performances of fencers in team competitions, with a specifical focus on world-class teams such as Ukraine, Estonia, Korea, and China during the 2016–2017 season. They calculated the effectiveness of each individual fencer by considering the cumulative points scored and received (i.e., positive or negative) and outlined five different approaches to team composition while accounting for each team member’s performance. For example, the team composition of Estonia remained stable during the season, with all participants adhering to an established sequence. In contrast, for China and Korea, the team composition was unstable, with the number/position changing based on the level of the competition, the team ranking and the composition of the opponent team.

With the aim of advancing our knowledge of the performance of elite female épée fencers, this study focused on individual events. Notably, for world championships, each country is limited to having only four fencers for each discipline (FIE, 2023). These fencers represent the best of their respective countries, making their performance particularly valuable for analysis and discussion. Therefore, we analyzed the performance of elite female épée fencers from the top 16 to final bouts in 2017–2019 world fencing championships, employing notational analysis. Various factors that impact how fencers score, such as techniques, piste areas, bout rounds, and score statuses were recorded per scoring action. The primary goal was to assess technique effectiveness in relation to these contextual factors. Moreover, we aimed to discern differences between winners and losers, with the objective of extracting insights from the performance of winners. The most relevant findings in match analysis would be a study by Aquili et al. (2013), which revealed that saber fencers executed attacks predominantly within the central 4-m zone. However, due to the rule differences between épée and saber, we expected that épée fencers might exhibit a preference not only for attacks, but also for counterattacks across a broader range of areas. Moreover, such a preference might interact with factors such as a bout round (1^st^, 2^nd^, and 3^rd^), a score status (leading, tied, or trailing), as well as the match result (winners and losers). The findings of this study are expected to provide valuable information to fencers, enabling them to make more informed tactical decisions, and to coaches, designing more effective training sessions as well as providing targeted feedback during bouts to enhance fencers’ performance (Hughes and Franks, 2007; O'Donoghue, 2014).

## Methods

### Sample

This study analyzed the performance of elite female épée fencers from top 16 to final bouts of the 2017, 2018, and 2019 world fencing championships. Earlier competitions before 2017 were not included due to differences in the non-combativity rule, which involved periods of 2 and 1 min without any touches being scored before and after 2017, respectively. Videos of the bouts were sourced from the official website of the International Fencing Federation (Fédération Internationale d'Escrime, FIE) on YouTube. The protocol was approved by the Institutional Review Board of the Fu Jen Catholic University, New Taipei, Taiwan (protocol code: C110181; approval date: 22 June 2022). In total, the dataset comprised 1,840 scoring events across 45 matches, involving 38 fencers.

### Design and Procedures

**Figure 1:**
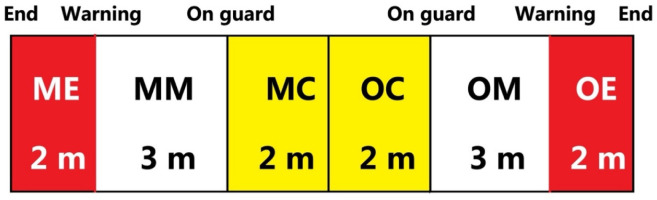
Areas on the fencing piste where fencers scored. Note: The areas were defined based on both fencers. For each fencer, the piste could be divided into 6 areas. ME, my own end area; MM, my own middle area; MC, my own central area; OC, opponent’s central area; OM, opponent’s middle area; OE, opponent’s end area

Each scoring event was notated based on the descriptive information of the bouts and the participating fencers (Table 1). This information included the year of the competition (2017, 2018 or 2019), the order of bouts (8 bouts of top 16, 4 bouts of top 8, 2 bouts of top 4, or 1 bout of top 2), and the specific round (1^st^, 2^nd^, 3^rd^, or the sudden death overtime). Details regarding the fencers, including their names and the side of the weapon arm (left or right), were recorded. The notations also encompassed the types of techniques employed, which included attack, riposte, counterattack or “others” encompassing the remise, redoublement, reprise of the attack, and counter-time, as per FIE technical rules (FIE, 2023). Specifically, the riposte, like the attack, is considered an offensive action, executed with the intention of hitting the opponent after parrying (i.e., deflecting) their attack. The counterattack comprises offensive or offensive-defensive actions made during the offensive action of the opponent, including an attack initiated against or into the opponent’s attack. The technique labeled as ‘others’ in this study included other offensive actions, including the remise (a renewal of an attack), the redoublement (a renewal of an action after being parried by replacing the point on the target in a different line to the original action), the reprise of the attack (a new attack executed immediately after a return to the on-guard position), and counter-time (any action made by the attacker against a stop hit made by the opponent). Moreover, the areas on the fencing piste where the fencers scored (central, middle, or end areas on the fencer’s own side or opponent’s side; as illustrated in Figure 1), the target position (inner or outer sides of the upper or the lower body), the outcome of the hit (single hit, double hit or no hit), the score status (leading, even or trailing), the score achieved (ranging from 0 to 15) for each scoring event, and the final outcome of the bout for the participating fencers (win or loss) were observed. The areas on the fencing piste where the fencers scored were determined by the middle point of the fencers’ two feet. The data regarding the side of the fencers’ weapon arm and the target position of hits were not analyzed in this study.

**Table 1:** List of notational analysis.

Item	Description
Year	2017, 2018, 2019
Order of bouts	Top 16 (1–8), Top 8 (1–4), Top 4 (1–2), Final
Round	1^st^, 2^nd^, 3^rd^, OT
Fencer	xxx
Weapon arm side	Left, Right
Piste area	ME, MM, MC, OC, OM, OE
Technique	Attack, Counterattack, Riposte, Others
Hit target	W arm side, Non-W side, Lower body
Hit result	None, Single, Double
Score achieved	0, 1, 2, 3, 4, 5, 6, 7, 8, 9, 10, 11, 12, 13, 14, 15
Score status	Leading , Even, Trailing
Bout result	Win, Loss

Note: OT, overtime; MC, my own central area; MM, my own middle area; ME, my own end area; OC, opponent’s central area; OM, opponent’s middle area; OE, opponent’s end area; W, weapon; Non-W, non-weapon

To ensure the reliability of the notations, a reliability test was conducted both within the operator (the first author of the study, aged 32, 20 years of épée fencing experience, participated in the 2012 London Olympic Games and 2006, 2010, and 2014 Asian Games, and held a domestic fencing referee certificate at the highest level) and between another operator (aged 30, 15 years of épée fencing experience, participated in the 2015 and 2017 Universiade Games and several times in Asian championships, and also held a domestic fencing referee certificate at the highest level). Both operators notated the final bout of the 2015 Moscow World Championships twice, with a two-week interval in between. As all the notations were nominal variables, their reliability was assessed using kappa statistic (Cohen, 1960; Hughes and Franks, 2007). The results revealed that all notations were consistent, with kappa values of 1.00, except for operator 1, where the kappa value was 0.90 for venue locations of the scoring actions. Therefore, the notations were considered to represent a very good level of agreement (Altman, 1991). Following the reliability test, the first author proceeded to complete the notations for all matches.

### Statistical Analysis

The notated data were then organized and analyzed as percentages of the total data. There were only 11 rounds with the sudden death overtime round among the 45 bouts, and therefore these data were not included in the analysis and discussion. For statistical analyses, we conducted two sets of three-way mixed-design ANOVAs, with the between-participants factor being whether fencers won or lost the bout (i.e., bout results). The within-participants factors in the 1^st^ set of analysis were hit results (single or double hits), while in the 2^nd^ set of analysis they were technique (attack, counterattack, riposte, and other techniques), both in conjunction with the factor of bout rounds (1^st^, 2^nd^, or 3^rd^). In the 3^rd^ set of analysis, we conducted a two-way (bout results x piste areas) mixed design ANOVA. For the 4^th^ and 5^th^ sets of data analysis, we divided the data based on bout results (i.e., winners and losers). We performed two-way repeated measures ANOVAs, one examining the factor of techniques scored and the other focusing on piste areas. These two sets of analyses were conducted based on the score status (leading, even or trailing). For all statistical analyses, we used IBM SPSS 26.0, with the alpha value set at 0.05. In situations where sphericity assumptions were violated, we applied Greenhouse Geisser correction. Effect sizes were calculated using partial eta-squared values, and for post hoc multiple comparisons, we employed Bonferroni's correction.

## Results

### Differences between Winners and Losers in Their Hit Results of Scoring Actions across Rounds

**Figure 2:**
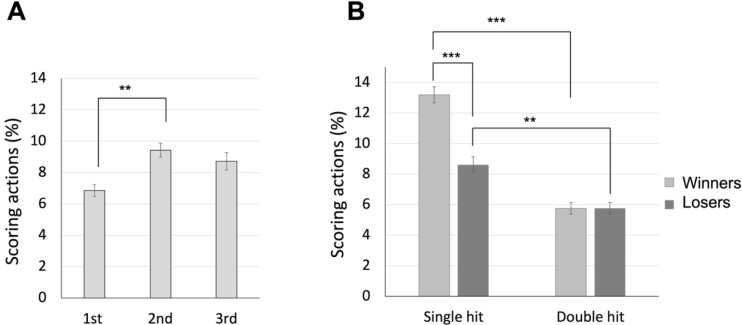
The average percentage of scoring actions across rounds (panel A); the average percentage of single hits and double hits for winners and losers (panel B). Note: * p < 0.05, **p < 0.005, *** p < 0.001

The 2 (bout results: winners vs. losers) x 2 (hit results: single hits vs. double hits) x 3 (rounds: 1^st^ vs. 2^nd^ vs. 3^rd^) ANOVA revealed significant main effects for all factors. Firstly, there was a significant main effect of bout results, F(1, 88) = 77.599, *p* < 0.001, *η_p_^2^* = 0.469, with the winners showing a higher percentage of scoring actions than losers (mean values of 9.48% and 7.19% for winners and losers, respectively). Secondly, there was a significant main effect of hit results, F(1, 88) = 74.031, *p* < 0.001, *η_p_^2^* = 0.457, with fencers achieving a greater percentage of single hits than double hits (mean values of 10.90% and 5.76% for single and double hits, respectively). The main effect of rounds was also significant, F(1.676, 147.512) = 5.967, *p* = 0.005, *η_p_^2^* = 0.064, with fencers showing a higher percentage of scoring actions in the 2^nd^ round than in the 1^st^ round (*p* < 0.001; mean values of 6.86%, 9.43%, and 8.72% for the 1^st^, 2^nd^, and 3^rd^ round, respectively; Figure 2A). Moreover, a significant bout results x hit results interaction was observed, F(1, 88) = 14.706, *p* < 0.001, *η_p_^2^* = 0.143 (Figure 2B). Post hoc analyses indicated winners achieved more single hits than losers (*p* < 0.001; mean values of 13.19% and 8.61% for winners and losers, respectively), while there were no significant differences in gaining double hits between the two groups (*p* = 1.000, mean values of 5.76% for both winners and lowers). On the other hand, both winners and losers had a greater percentage of single hits than double hits (*p* values < 0.005). The other interaction effects were not significant: bout results x rounds interaction, F(2, 176) = 0.419, *p* = 0.658, *η_p_^2^* = 0.005; hit results x rounds interaction, F(2, 176) = 2.793, *p* = 0.064, *η_p_^2^* = 0.031; and three-way interaction, F(2, 176) = 0.691, *p* = 0.539, *η_p_^2^* = 0.007.

### Differences between Winners and Losers in Performing Various Scoring Techniques across Rounds

**Figure 3:**
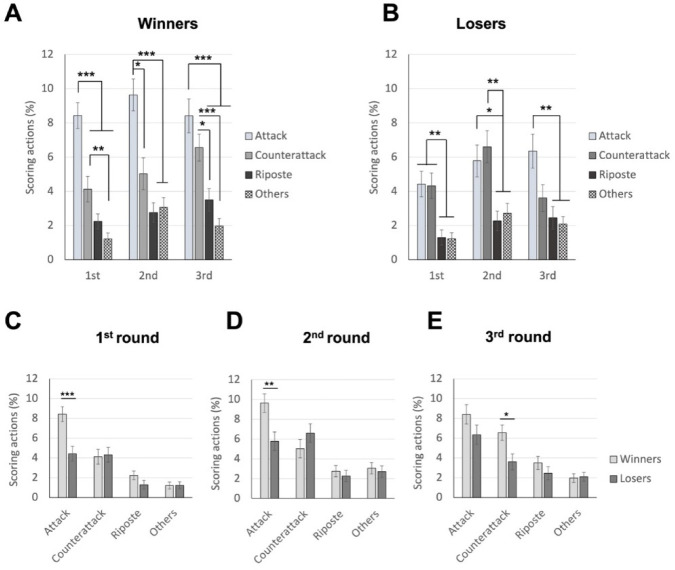
The average percentage of scoring techniques for winners and losers across rounds; top panels demonstrate data for winners (panel A) and losers (panel B), respectively; bottom panels demonstrate data for 1^st^ (panel C), 2^nd^ (panel D), and 3^rd^ (panel E) rounds, respectively. Note: * p < 0.05, ** p < 0.005, *** p < 0.001

The 2 (bout results: winners vs. losers) x 4 (techniques: attack vs. counterattack vs. riposte vs. others) x 3 (rounds: 1^st^ vs. 2^nd^ vs. 3^rd^) ANOVA also revealed significant main effects for all factors. The main effects of bout results, F(1, 88) = 77.836, *p* < 0.001, *η_p_^2^* = 0.469, and rounds, F(1.699, 149.531) = 6.220, *p* = 0.004, *η_p_^2^* = 0.066, replicated the result of 1^st^ set of analysis. The main effect of technique was also significant, F(2.361, 207.732) 47.513, *p* < 0.001, *η_p_^2^* = 0.351, with fencers scoring through attacks most frequently, followed by counterattacks, and then ripostes and other techniques (mean values of 7.17%, 5.04%, 2.42% and 2.04% for attack, counterattack, riposte, and other techniques, respectively). Pairwise differences were significant (*p* < 0.01) except for the one between riposte and other techniques (*p* = 1.000). Moreover, the three-way interaction was significant, F(6, 528) = 2.403, *p* = 0.027, *η_p_^2^* = 0.027. As shown in Figure 3A, post hoc analyses indicated that for the four scored techniques, winners showed the same trend as in the main effect of techniques in the 1^st^ round (mean values of 8.42%, 4.12%, 2.24% and 1.21% for attack, counterattack, riposte, and other techniques, respectively), with all pairwise comparisons significant except for the one between riposte and others (*p* = 0.555) and additionally between the counterattack and the riposte (*p* = 0.326). In the 2^nd^ round, the trend was also similar (mean values of 9.63%, 5.02%, 2.75% and 3.06% for attack, counterattack, riposte, and other techniques, respectively), with all pairwise comparisons significant except for the one between the riposte and others (*p* = 1.000), and between the counterattack and the riposte (*p* = 0.383) as well as others (*p* = 0.506). However, in the 3^rd^ round, winners scored through counterattacks more frequently (mean values of 8.40%, 6.65%, 3.50% and 1.97% for attack, counterattack, riposte, and other techniques, respectively), with no significant differences between the attack and the counterattack (*p* = 0.816). The riposte and other techniques were still scored the least, with no differences between them (*p* = 0.243). As shown in Figure 3B, contrastingly, in the 1^st^ and 2^nd^ rounds, losers showed a similar trend but did not score differently between the attack and the counterattack (*p* = 1.000) as well as between the riposte and other techniques (*ps* = 1.000) (mean values of 4.42%, 4.32%, 1.30% and 1.22% for the four techniques in the 1^st^ round, and mean values of 5.78%, 6.60%, 2.27% and 2.71% for the four techniques in the 2^nd^ round). In the 3^rd^ round, the mean values for attack, counterattack, riposte, and other techniques were of 6.34%, 3.60%, 2.45% and 2.09%, respectively. Significant differences were only found in the attack compared to the riposte (*p* = 0.008) and other techniques (*p* = 0.001).

As shown in Figure 3C and Figure 3D, notably, winners scored via attacks more frequently compared to losers in the 1^st^ and 2^nd^ rounds (*p* values < 0.005; mean values of 8.42% and 4.42% for winners and losers, respectively, in the 1^st^ round; and mean values of 9.63% and 5.78% for winners and losers, respectively, in the 2^nd^ round). In the 3^rd^ round, winners scored more frequently than losers using counterattacks (*p* = 0.010; mean values of 6.55% and 3.60% for winners and losers, respectively; Figure 3E). Lastly, for losers, the round differences were found between the 2^nd^ and 3^rd^ rounds for the counterattack (*p* = 0.035; mean values of 4.32%, 6.60% and 3.60% for the three rounds, respectively), whereas for winners, it was between the 1^st^ and 2^nd^ rounds for other techniques (*p* = 0.011; mean values of 1.21%, 3.06% and 1.97% for the three rounds, respectively). The bout results x techniques interaction was significant, F(3, 264) = 4.445, *p* = 0.005, *η_p_^2^* = 0.048; the other interaction effects were not significant: bout results x rounds interaction, F(2, 176) = 0.420, *p* = 0.658, *η_p_^2^* = 0.005; techniques x rounds interaction, F(6, 528) = 0.439, *p* = 0.853, *η_p_^2^* = 0.005.

### Differences of Piste Areas where Winners and Losers Scored

**Figure 4:**
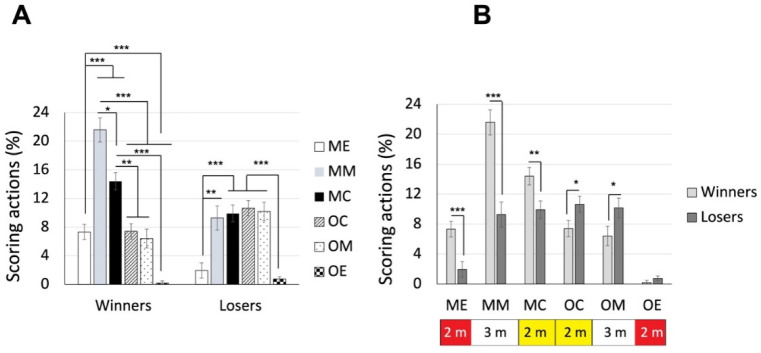
The average percentage of piste areas where winners and losers scored. Note: The areas were defined based on both fencers. For each fencer, the piste could be divided into 6 areas. ME, my own end area; MM, my own middle area; MC, my own central area; OC, opponent’s central area; OM, opponent’s middle area; OE, opponent’s end area. * p < 0.05, ** p < 0.005, *** p < 0.001

The two (bout results: winners vs. losers) x six (piste areas: ME vs. MM vs. MC vs. OC vs. OM vs. OE) ANOVA revealed a significant interaction effect, F(5, 440) = 11.667, *p* < 0.001, *η_p_^2^* = 0.117. As shown in Figure 4A, post hoc analysis indicated that for winners, the middle area of their own side was where they scored most frequently, followed by the central area of their own side, then central and middle areas of the opponent’s side and the end area of their own side, finally the end area of the opponent’s side (mean values of 7.32%, 21.59%, 14,40%, 7.40%, 6.41%, and 0.18% for ME, MM, MC, OC, OM, and OE areas, respectively). Whereas for losers, the four areas in the central to middle areas were where they scored the most, followed by the end areas of both sides (mean values of 1.94%, 9.28%, 9.90%, 10.64%, 10.18%, and 0.75% for ME, MM, MC, OC, OM, and OE areas, respectively). On the other hand, the group differences were found in all piste areas (*p* values < 0.05) except for the opponent’s end area (*p* = 0.223) (Figure 4B). The main effects of both bout results, F(1, 88) = 97.184, *p* < 0.001, *η_p_^2^* = 0.525, and piste areas, F(3.214, 282.832) = 34.665, *p* < 0.001, *η_p_^2^* = 0.283, were significant.

### Differences between Winners and Losers in Performing Various Scoring Techniques Based on the Score Status (Leading, Even or Trailing)

As we already learned from previous analysis that the four techniques were scored differently by winners and losers, in this set of analysis we examined their respective data separately based on different score statuses. Therefore, two separate two-way (4 techniques x 3 score statuses) ANOVAs were conducted for winners and losers.

**Figure 5:**
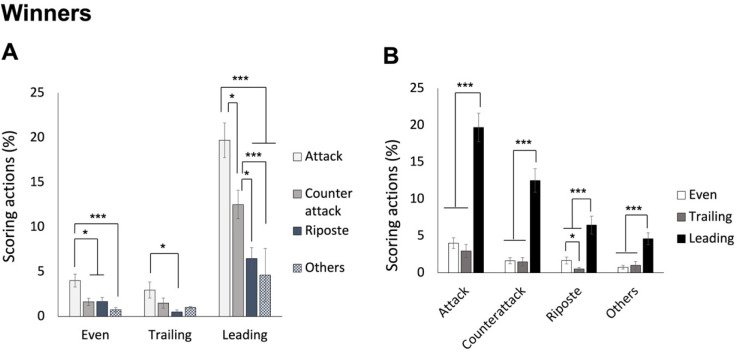
The average percentage of scoring techniques based on different score statuses for winners. Panel A shows the technique differences in the three score statuses; panel B shows the status differences for the four techniques. Note: * p < 0.05, ** p < 0.005, *** p < 0.001

For winners, ANOVA revealed a significant interaction effect, F(3.048, 134.110) = 12.302, *p* < 0.001, *η_p_^2^* = 0.218. As shown in Figure 5A, post hoc analyses indicated that when leading, winners scored through attacks most frequently, followed by counterattacks, and then ripostes and other techniques (mean values of 19.70%, 12.51%, 6.46%, and 4.62%, for attack, counterattack, riposte, and other techniques, respectively). While the score was even, the attack was still scored most frequently, followed by all the other three types of techniques (mean values of 4.01%, 1.63%, 1.67%, and 0.76%, for attack, counterattack, riposte, and other techniques, respectively). When winners were trailing in scores, the attack was still the most performed scoring action, but it was the riposte that was the least used (mean values of 2.96%, 1.49%, 0.51%, and 1.01%, for attack, counterattack, riposte, and other techniques, respectively). On the other hand, as shown in Figure 5B, the differences between leading and the other two score statuses were significant for all techniques (*p* values < 0.005), and specifically for the riposte, the differences between even and trailing score conditions were also significant (*p* = 0.037).

**Figure 6:**
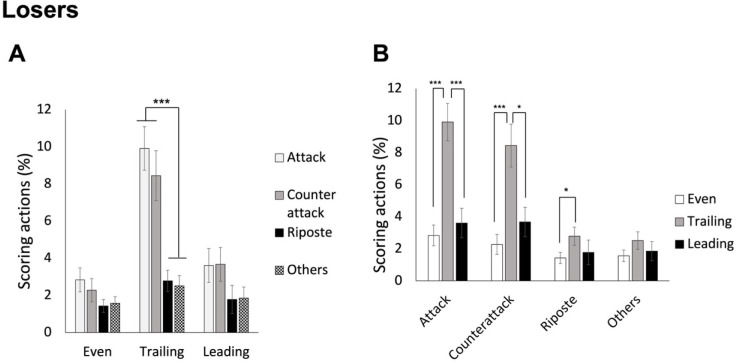
The average percentage of scoring techniques based on different score statuses for losers. Panel A shows the technique differences in the three score statuses; panel B shows the status differences for the four techniques. Note: * p < 0.05, ** p < 0.005, *** p < 0.001

For losers, ANOVA also revealed a significant interaction effect, F(3.911, 172.104) = 5.984, *p* < 0.001, *η_p_^2^* = 0.120. As shown in Figure 6A, post hoc analyses indicated that when both leading and tied, losers did not score with any specific technique more frequently (mean values of 3.61%, 3.67%, 1.78%, and 1.86%, for attack, counterattack, riposte, and other techniques, respectively, in leading status; mean values of 2.84%, 2.76%, 1.43%, and 1.57%, for the four techniques, respectively, in even status). Instead, when losers were trailing in scores, the attack and the counterattack were scored more frequently than the other two techniques (mean values of 9.91%, 8.44%, 2.79%, and 2.52%, for attack, counterattack, riposte, and other techniques, respectively). On the other hand, as shown in Figure 6B, the differences between trailing and the other two score statuses were significant for the attack and the counterattack (*p* < 0.05), and specifically for the riposte, the differences between an even and a trailing status were significant (*p* = 0.02).

### Differences where Winners and Losers Scored in Piste Areas Based on the Score Status (Leading, Even or Trailing)

**Figure 7:**
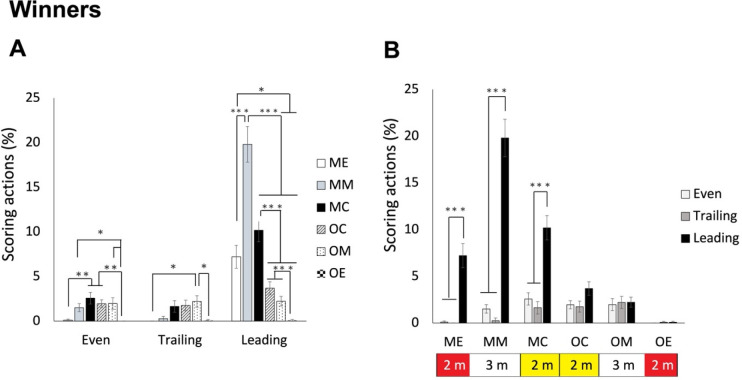
The average percentage of piste areas where winners scored in different score statuses. Panel A shows the area differences in the three score statuses; panel B shows the status differences for the 6 areas. Note: The areas were defined based on both fencers. For each fencer, the piste could be divided into 6 areas. ME, my own end area; MM, my own middle area; MC, my own central area; OC, opponent’s central area; OM, opponent’s middle area; OE, opponent’s end area. * p < 0.05, ** p < 0.005, *** p < 0.001

As for the 4^th^ set of analysis, we examined data of winners and losers separately based on a different score status. Therefore, two separate two-way (6 piste areas x 3 score statuses) ANOVAs were conducted for winners and losers. For winners, ANOVA revealed a significant interaction effect, F(3.784, 166.510) = 31.921, *p* < 0.001, *η_p_^2^* = 0.420. As shown in Figure 7A, post hoc analyses indicated that when leading, winners scored in their own middle area most frequently, followed by their end and central areas, and then the opponent’s central and middle areas, finally the opponent’s end area (mean values of 7.21%, 19.81%, 10,19%, 3.68%, 2.23%, and 0.09% for ME, MM, MC, OC, OM, and OE areas, respectively). When the score was even, they scored in the middle and central areas of both sides more frequently than in the end areas of both sides (mean values of 0.11%, 1.52%, 2,58%, 1.96%, 1.98%, and 0.00% for ME, MM, MC, OC, OM, and OE areas, respectively). When they were trailing, the opponent’s middle area was where they scored more frequently than the end areas of both sides (*p* values < 0.05; mean values of 0.00%, 0.27%, 1.64%, 1.76%, 2.21%, and 0.09% for ME, MM, MC, OC, OM, and OE areas, respectively). On the other hand, as shown in Figure 7B, the differences between leading and the other two statuses for their own sides (including end, middle and central areas) were significant (*p* values < 0.005), whereas the differences among all statuses for the opponent’s side were all not significant (*p* values > 0.146).

**Figure 8:**
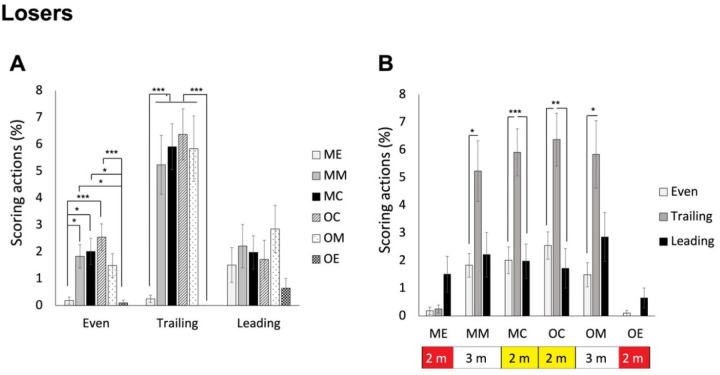
The average percentage of piste areas where losers scored in different score statuses. Panel A shows the area differences in the three score statuses; panel B shows the status differences for the 6 areas. Note: The areas were defined based on both fencers. For each fencer, the piste could be divided into 6 areas. ME, my own end area; MM, my own middle area; MC, my own central area; OC, opponent’s central area; OM, opponent’s middle area; OE, opponent’s end area. * p < 0.05, ** p < 0.005, *** p < 0.001

For losers, ANOVA revealed a significant interaction effect, F(4.273, 188.006) = 4.457, *p* < 0.001, *η_p_^2^* = 0.092. As shown in Figure 8A, post hoc analyses indicated that when the score was even, fencers scored in the middle and central areas of both sides more frequently than in the end areas of both sides (mean values of 0.19%, 1.83%, 2,01%, 2.54%, 1.49%, and 0.10% for ME, MM, MC, OC, OM, and OE areas, respectively). A similar trend was found when they were trailing (*p* < 0.001; mean values of 0.25%, 5.24%, 5.91%, 6.37%, 5.84%, and 0.00% for ME, MM, MC, OC, OM, and OE areas, respectively). Instead, whey they were leading, there were no differences among the areas (mean values of 1.51%, 2.21%, 1,98%, 1.72%, 2.85%, and 0.65% for ME, MM, MC, OC, OM, and OE areas, respectively). On the other hand, as shown in Figure 8B, the differences between trailing and the even status were significant for central and middle areas of both sides (*p* values < 0.01), additionally with significant differences between trailing and leading areas for central areas of both sides (*p* values < 0.005), whereas the differences among all statuses for the end areas of both sides were all not significant (*p* values > 0.083).

## Discussion

This study focused on analyzing the performance of elite female épée fencers during individual events in world championships. We systematically examined how various contextual factors, including piste areas, bout rounds, and score statuses, influenced the use of techniques, scoring outcomes and the eventual bout outcomes. The 1^st^ set of analysis revealed that the 2^nd^ round was the most crucial round, with fencers achieving more hits compared to the 1^st^ round. The lower number of hits in the 1^st^ round could be attributed to a cautious and conservative approach adopted by both fencers in a process of probing and familiarizing themselves with the opponent’s movements and tactics. As they progressed into the 2^nd^ round and gained a better understanding of each other’s playing style, they tended to become more assertive and actively engage in actions aimed at scoring points. Notably, winners demonstrated a higher proficiency in scoring single hits than losers. Notably, both winners and losers scored more single hits than double hits, underscoring the importance of single hits in determining the bout’s outcome even in épée where double hits are allowed (Roi and Bianchedi, 2008).

Which techniques are crucial for scoring? While in saber fencing, the majority of actions are offensive (including attacks and remise) (Aquilli et al., 2013), we expected that épée fencers might demonstrate a preference not only for attacks but also for counterattacks, and this preference might interact with other factors such as the match result (winner and loser). The 2^nd^ set of analysis revealed that, as saber fencers (Aquilli et al., 2013), winners consistently scored more points with attacks than the other three types of techniques across the three rounds, whereas losers, having a lower number of attacks in the first two rounds, gained more points from both attacks and counterattacks compared to the other two types of techniques, as we expected. On the other hand, for 1^st^ and 2^nd^ rounds, winners differentiated themselves with losers by scoring a higher number of hits using attacks. Interestingly, in the 3^rd^ round, winners achieved more hits through counterattacks compared to losers. The 4^th^ set of analysis further indicated that regardless of whether winners were leading, tied or trailing in scores, they utilized attacks more frequently than the other techniques to score. Losers, on the other hand, only showed this trend (utilizing attacks more than ripostes and other techniques) when they were trailing in scores. These findings suggest that winners intentionally performed attacks to score across all rounds consistently and under different score conditions. By accumulating more points from the first two rounds than losers, they were then able to effectively employ counterattacks in the 3^rd^ round. This strategic approach could offer valuable insights for managing different rounds of a bout.

How to effectively utilize the piste area? While saber fencers score most frequently in the central 4 m (that is two central areas) of the piste (Aquilli et al., 2013), we expected that épée fencers might utilize the piste across a wider range of areas. Indeed, the 3^rd^ set of analysis revealed that losers scored more frequently in the middle four areas (middle and central areas of both sides) than in the two end areas. In contrast, winners scored more frequently in their own middle and central areas than in the end area of the opponent’s side. Interestingly, the lower percentage of scoring in the end area of their own side might be attributed to the pressure of potentially exiting the piste in that area. When comparing winners and losers in each area, we found that winners scored more frequently in their own side than losers. Instead, losers scored more frequently in the opponent’s central and middle areas. This strategic use of the piste area might be specific to épée, which preserves the original dueling nature by allowing double hits and does not consider the priority rule. The 5^th^ set of analysis further indicated that winners and losers scored in the piste areas differently under various score conditions. Winners scored more frequently in their own side of the piste when leading. They showed the tendency of scoring in the central areas of both sides more frequently than in the end areas of both sides when temporarily tied. When temporarily trailing in scores, they scored more in the middle area of the opponent’s side. These results indicated that épée fencers became more aggressive when they were not leading in scores. On the contrary, regardless of trailing or being tied, losers scored more frequently in the central and middle areas of both sides compared to the two end sides. It might reflect that when fencers were temporarily leading, they tended not to actively move forward but instead probed or waited for the opponent to advance to their side, allowing them to find opportunities to perform scoring actions. Moreover, losers did not exhibit a preference for specific piste areas while temporarily leading in scores. These result highlight that épée fencing does not necessarily require fencers to be aggressive in pushing the opponent to the opponent’s side of the piste all the time. Attracting the opponent to one’s own side, particularly the middle and central areas, and finding the opportunity to execute attacks (in the first two rounds) and counterattacks (in the 3^rd^ round) could be more effective, whereas when temporarily trailing or tied, being slightly more aggressive in pushing the opponent to the central areas or even further to the middle area of the opponent’s side is recommended.

## Conclusions

Overall, our results demonstrate that in épée fencing the use of techniques is shaped by various contextual factors, which serve as task constraints (Newell, 1986). The 2^nd^ round was an important scoring round, probably following a process of probing and familiarization with the opponent’s movement and tactics in the 1^st^ round. Winners employed a strategic approach that prioritized a higher number of single hits, particularly through attacks in the first two rounds and counterattacks in the 3^rd^ round. Additionally, winners aimed to lure the opponent toward their own side of the piste when they were leading in scores. However, in situations where they were temporarily tied or trailing, a slightly more aggressive approach of advancing into the central areas or even the opponent’s middle area to score using attacks was applied. In contrast, in these situations, losers frequently scored in the central and middle areas of both sides. They showed no preference for techniques or piste areas when temporarily leading. Our findings provide valuable insights for coaches to design conditioned training programs. For example, following the constraint-led approach (Chow, 2013; Renshaw and Chow, 2019), coaches can set specific objectives for fencers. In the 1^st^ round, they might restrict fencers to using only attacks for scoring or incentivize increased use of attacks by doubling the points awarded. Similarly, coaches can simulate scenarios in which one fencer is leading in scores during the 3^rd^ round, offering doubled points for scoring a single hit with a counterattack or within the middle and central areas of the fencer’s own side. Moreover, the insights from our study can help coaches offer targeted feedback to fencers during specific bouts to enhance fencers’ performance.
